# Successful use of combined blood purification techniques in splenectomised patient with septic shock in streptococcus pneumoniae infection – a case report

**DOI:** 10.1186/s12879-018-3327-y

**Published:** 2018-08-29

**Authors:** Andreja Sinkovic, Barbara Kit, Andrej Markota

**Affiliations:** Department of medical intensive care medicine, University clinical centre Maribor, Ljubljanska 5, 2000 Maribor, Slovenia

**Keywords:** Sepsis, Septic cardiomyopathy, Septic shock, Combined blood purification techniques

## Abstract

**Background:**

Septic cardiomyopathy represents cardiac impairment in sepsis and is a part of systemic involvement in sepsis. Cytokine storm is responsible for septic shock and for myocardial dysfunction of potentially reversible septic cardiomyopathy. Several case reports and case series demonstrated successful removal of circulating cytokines by combined blood purification techniques. In this way, septic shock and survival of septic patients improved. However, the evidences for reversal of myocardial dysfunction are rare.

**Case presentation:**

We present a patient with a history of chemotherapy for coat cell lymphoma, splenectomy and autologous bone marrow transplantation, who suffered severe pneumococcal sepsis, septic shock and septic cardiomyopathy, resistant to pharmacological therapy. Combined blood purification techniques 36 h after the start of treatment successfully decreased Interleukin-6 level, lactacidosis, the need for vasopressors to maintain normotension, improved systolic function of the left ventricle and clinical outcome.

**Conclusions:**

Our case suggests that combined blood purification techniques initiated even 36 h after the start of treatment successfully removed inflammatory cytokines, reversed circulatory failure and improved left ventricular systolic function in pneumococcal sepsis.

## Background

Septic cardiomyopathy presents cardiac dysfunction as a part of systemic involvement in sepsis [1–3]. It is acute and completely reversible, if the patients survive. It still is a condition of high prognostic importance because it accounts for approximately 10% of the fatalities observed in sepsis and septic shock [1–3]. Some data suggest, that nonsurvivors have more depressed afterload-related myocardial dysfunction than survivors have [1].

Septic cardiomyopathy occurs in up to 65% of septic patients, according to echocardiographic measurements of ejection fraction (EF). Depressed LV systolic function in septic cardiomyopathy is associated with normal or low left ventricular filling pressure, unlike the pattern of cardiogenic shock, where the left ventricular pressures are elevated [2].

In sepsis with significant afterload reduction (because of decreased systemic vascular resistance), left and right ventricular cardiac output and stroke volumes are not adequately increased or are even depressed. In septic cardiomyopathy, we can find global and/or regional contractile disturbances as well as diastolic dysfunction. Increased left ventricular compliance with a shift of the pressure-volume curve to the left causes considerable dilation of the left and right heart, which is a prognostically positive sign, according to some studies [1, 2].

Acute systolic myocardial dysfunction of the left ventricle in cases with septic cardiomyopathy contributes significantly to hypotension in septic shock [1–3].

Circulating factors responsible for systolic myocardial dysfunction in sepsis are toxins, cytokines, cardio depressant factors, etc. Complement activation and apoptosis are important as well. Among cardio depressant cytokines TNF-α increases intracellular nitric oxide, mitochondrial dysfunction of the cardiomyocytes and even phosphorylation of troponin I and thus reducing cardiac myofilament response to Ca^2+^ [1, 2]. Proinflammatory cytokines depress myocardial function, but also stimulate cytokine production in the heart, in particular IL-6 [1, 2, 4, 5]. Cytokine storm in sepsis, in particular the release of IL-6, results in hemodynamic instability in septic shock [6].

In animal and human studies, removal of circulating cytokines by blood purification techniques improves hemodynamic status and survival in sepsis [7–9].

Several case reports and case series demonstrated successful removal of circulating cytokines by hemoadsorption membranes such as CytoSorb® [9, 10]. However, the evidence for reversal of systolic myocardial dysfunction by blood purification technique combined with hemoadsoption, are rare. Our purpose was to demonstrate the efficacy of cytokine removal by combined use of continuous veno-venous hemofiltration (CVVH) and hemoadsorption by CytoSorb® in treatment of a patient with septic shock and septic cardiomyopathy, resistant to vasopressors and inotropes.

## Case presentation

52-year old woman with a history of chemotherapy for coat cell lymphoma in 2011, splenectomy in 2013 and autologous bone marrow transplantation in 2014 was admitted to the medical intensive care unit (ICU) after having fever up to 38.7 °C and malaise for 24 h. On admission, she was somnolent; the skin was cold, wet and pale; body temperature was 38 °C, blood pressure 50/40 mmHg and puls 120/min. She was eupnoeic with oxygen saturation (SatO2) of 100% by pulse oximetry, inspiring 2 L of oxygen by nasal cannula. Clinical examination revealed rales over both lungs and tachycardia without heart murmurs. Abdomen was soft and painless with audible peristalsis. Standard electrocardiogram (ECG) showed sinus tachycardia of 125/min.

On admission, we started continuous ECG monitoring, pulse oximetry, non-invasive blood pressure measurements and inserted central venous, arterial and urine catheters to measure central venous pressure intermittently, arterial blood pressure continuously and diuresis per hour.

We suspected sepsis with septic shock and immediately started treatment of shock and diagnostic procedures for sepsis. We managed shock initially by rapid infusion of crystalloids until we confirmed fluid unresponsiveness by ultrasound of inferior vena cava, demonstrating its diameter of 2.2 cm, that did not change with inspiration. Therefore, we started noradrenalin infusion within the first 15 min and up titrated it to 66μg/min. In addition, bedside echocardiography showed decreased ejection fraction (EF) of the left ventricle to 20%. We added dobutamine infusion, but also glucocorticoids and later on vasopressin to reach normotension.

From the very start we suspected pneumonia on clinical grounds and confirmed it by bilateral infiltrates on chest rentgenograph. Among admission laboratory data we observed lactacidosis (arterial pH 7.24, bicarb 13.4 mmol/l, pCO_2_ 4.24 kPa, pO_2_ 13 kPa, lactate 7.5 mmol/l), thrombocytopenia (62 × 10^3^/μL), leucocytosis, increase of procalcitonin to 100 ng/ml, C-reactive protein (CRP) to 166 mg/l, N-terminal-pro brain natriuretic peptide (NT-proBNP) to 2114 pmol/l, myoglobin to 482μg/l, and serum creatinine to 288 μg/l. Admission SOFA score was eight. We collected hemocultures, urinoculture and aspirates as soon as possible and after that immediately administered imipenem 500 mg/6 h IV.

After the first 24 h positive pneumococcal urine antigen confirmed streptococcal pneumonia. We continued imipenem therapy and adjusted the dose to renal failure. Other microbiological cultures remained negative. Together with the specialist for infectious disease we decided to continue imipenem therapy due to prior disease, including splenectomy.

After 24 h of ICU-stay the patient needed 40% oxygen by mask to achieve satisfactory blood gases (pH 7.2, bicarb 15 mmol/l, paCO_2_ 5.35 kPa, paO_2_ 8.5 kPa), her body temperature was 38 °C. SatcvO_2_ was 76.1%. Luckily, the patient did not need neither non-invasive, nor invasive ventilation during the entire ICU stay.

In spite of all treatments, after the first 24 h multiorgan failure syndrome persisted, including severe systolic myocardial dysfunction with left ventricular EF of 20%, measured by echocardiography. SOFA score at that time was 12.

After 36 h of ICU stay resistant septic shock with high-dose catecholamine support, left ventricular dysfunction with EF of 20% persited and renal failure (serum creatinine 379μmol/l, daily urine output < 500 ml) worsened. SatcvO2 was 78%, body temperature 37 °C and SOFA score increased to 13.

In addition to echocardiography, Pulse Contour Cardiac Output (PiCCO) catheter was inserted to improve hemodynamic monitoring and demonstrated cardiac index (CI) of 3.3 l/min/m^2^) with stroke volume (SV) of 50 ml, increased global end-diastolic index (GEDI) to 1023 ml/m^2^ and extra vascular lung water index (ELWI) to 13.3 ml/kg and decreased systemic vascular resistance index (SVRI) of 1672 dyn.s.cm^− 5^.m^2^.

Persistant hemodynamic instability and worsening renal failure led to the decision to start continuous veno-venous hemofiltration (CVVH) combined with hemoadsoption treatment by CytoSorb® membrane for the next 24 h. The goal was to improve hemodynamic situation and modulate the inflammatory response in our splenectomised septic patient. Before the start of blood purification therapy, we measured serum IL-6 level, which was 114 pg/ml.

After only 24 h of CVVH with concomitant use of a single CytoSorb® membrane EF increased to 45%. PiCCO measurements improved as follows: GEDI changed to 805 ml/m^2^, ELWI to 11.2 ml/kg, SVR to 1888 dyn.s.cm^− 5^.m^2^ and CI to 3.95 min/m^2^ and SV to 61 ml. The patient’s temperature was 37 °C and SOFA score 11. IL-6 dropped from 114 pg/ml to 14,2 pg/ml after termination of hemoadsoption therapy.

**Fig. 1:**
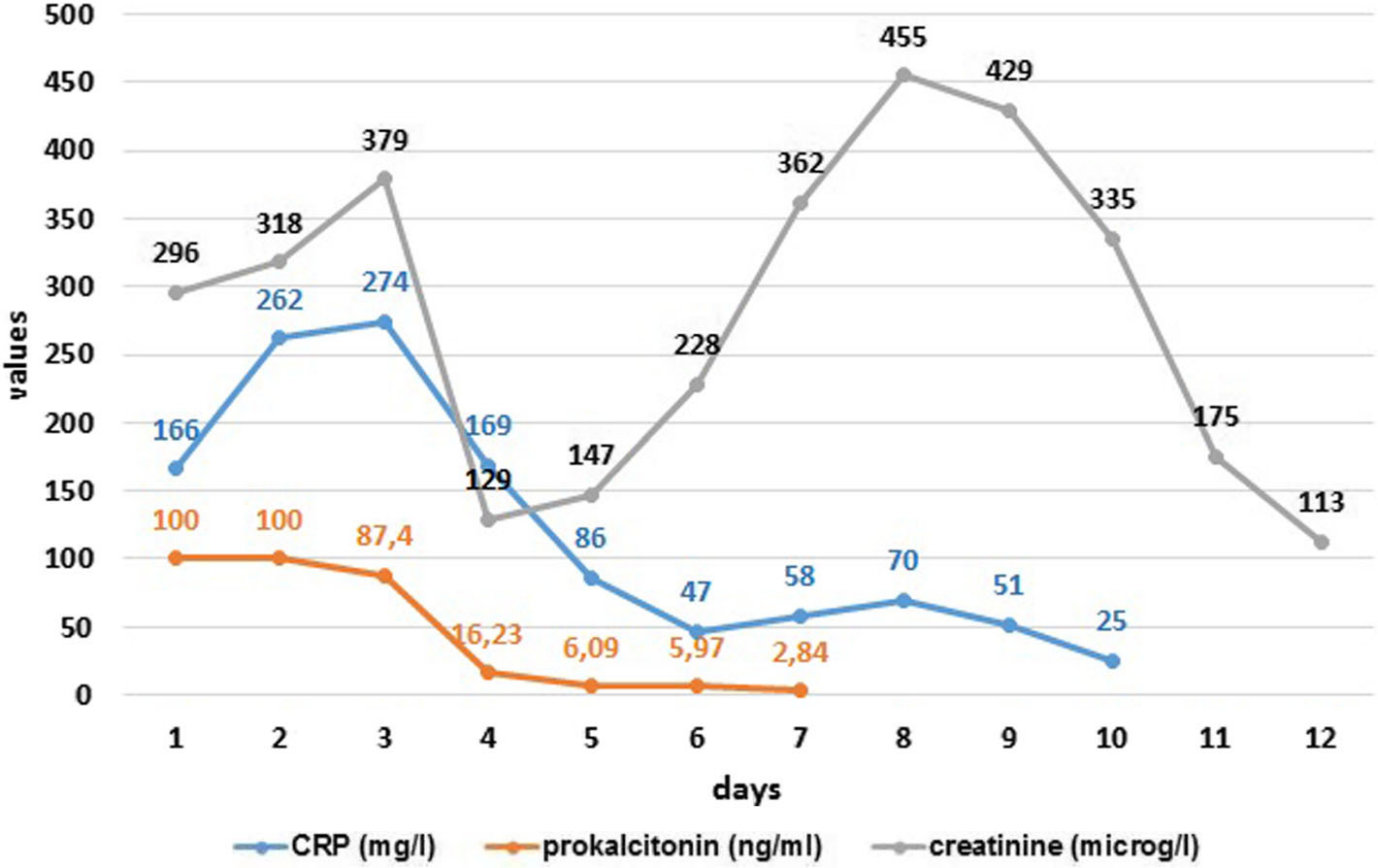
Changes in CRP, procalcitonin and serum creatinine.

**Fig. 2:**
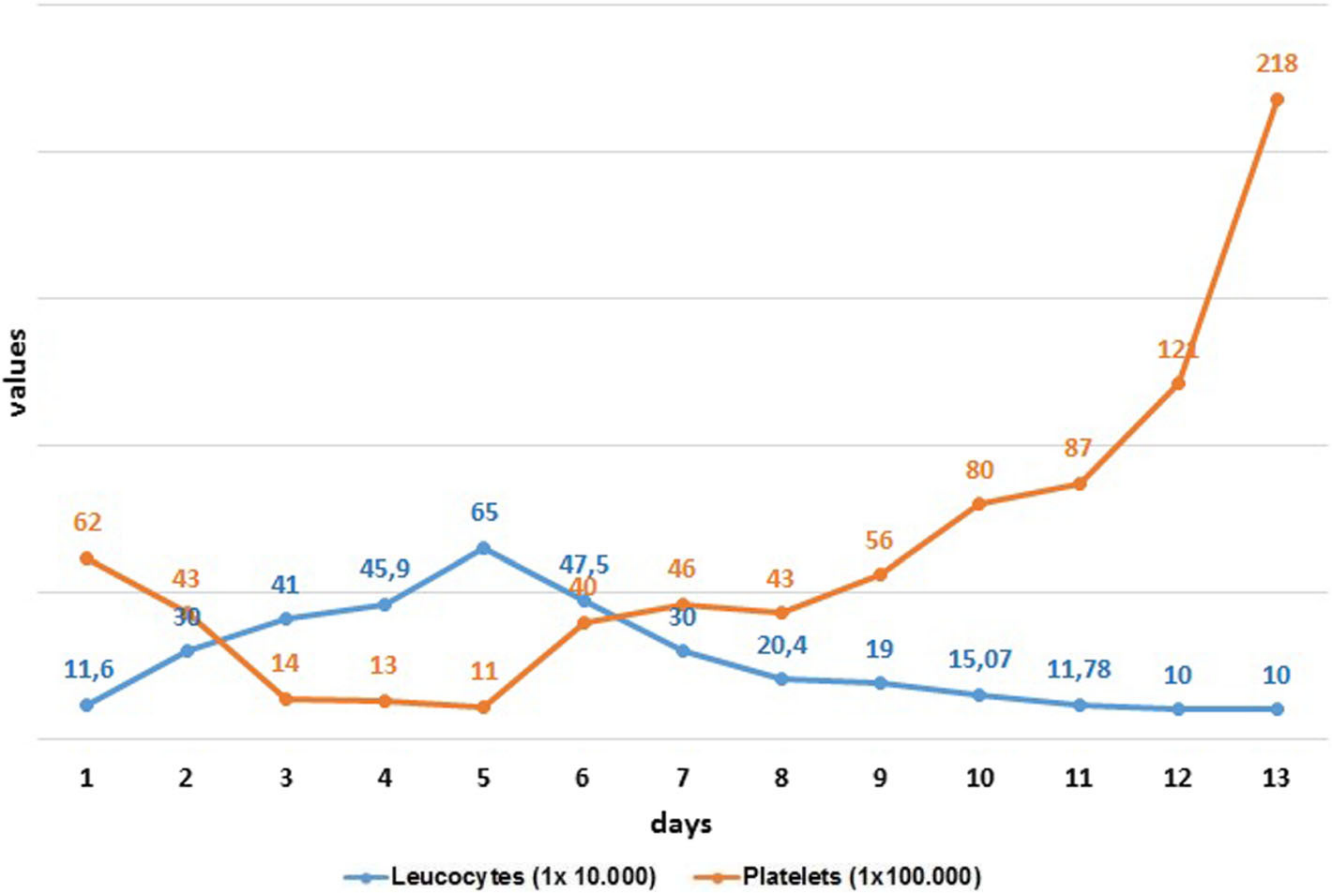
Changes in leucocytes and platelets.

We could stop the use of dobutamine, norepinephrine and vasopressin. The next day SOFA score was seven. Serum lactate and arterial pH turned to normal within few days, as well as CRP, procalcitonin (Fig. 1), leucocyte and platelet count after 14 days (Fig. 2). Table 1 presents the course of the treatment.

**Table 1:** Time line table

Date	Relevant past medical history and interventions		
2011	coat cell lymphoma diagnosed and chemotherapy performed		
2013	splenectomy performed		
2014	autologous bone marrow transplantation performed		
Dates	Summaries from initial and follow-up visits	Diagnostic testing, including dates	Interventions
10.10. 2015	-Initial visit (admission to ICU): After 24-h of fever and malaise the patient was admitted somnolent, breathing spontaneously with cold, wet and pale skin, 38 °C of fever; blood pressure 50/40 mmHg, puls 120/min, eupnoeic, with rales over both lungs, without heart murmurs, soft and painless abdomen with audible peristalsis. -After 30–45 min of therapy: Oriented, breathing spontaneously, blood pressure was 110/65 mmHg, puls 120/min -After 2 h of therapy: Oriented, breathing spontaneously, blood pressure was 100/60 mmHg to 120/80 mmHg, puls 120/min, diuresis 20/min, SOFA score 8	-pulse oximetry -standard ECG -continuous ECG monitoring -insertion of central venous, arterial and urine catheters to measure arterial blood pressure continuously, central venous pressure intermittently and urine output per hour -ultrasound of vena cava inferior and EF -laboratory tests -Chest roentgenogram -microbiological cultures	−2 L of oxygen by nasal cannula. -rapid infusion of crystalloids, -noradrenalin iv. infusion -dobutamine iv. -glucocorticoids iv. -vasopressin - Imipenem iv.
11.10.2015	Oriented, breathing spontaneously, blood pressure was 110/60 mmHg to 120/60 mmHg, puls 140–100/min, diuresis 225 ml/24 h, SOFA score 12	-pulse oximetry -continuous ECG, arterial blood pressure, intermittent central venous pressure monitoring and hourly urine output -ultrasound of vena cava inferior and EF -laboratory tests -Chest roentgenogram -PiCCO catheter insertion and hemodynamic measurements	−40% of oxygen by mask -infusion of crystalloids, noradrenalin, dobutamine, vasopressin -glucocorticoids iv. -imipenem iv.
12.10.2015	Oriented, breathing spontaneously, blood pressure was 100/60 mmHg to 120/80 mmHg, puls 130–100/min, diuresis 225 ml/24 h, SOFA score 13	-pulse oximetry -continuous ECG, arterial blood pressure, intermittent central venous pressure monitoring and hourly urine output -ultrasound of vena cava inferior and EF -laboratory tests -Hemodynamic measurements by PiCCO catheter	−40% of oxygen by mask -infusion of crystalloids, noradrenalin, dobutamine, vasopressin -glucocorticoids iv. -imipenem iv. -CVVH and CytoSorb® started
13.10.2015	Oriented, breathing spontaneously, afebrile, blood pressure was 120/60 mmHg to 120/80 mmHg, puls 130–100/min, diuresis 200 ml/24 h, SOFA score 11	-pulse oximetry -continuous ECG, arterial blood pressure, intermittent central venous pressure monitoring and hourly urine output -ultrasound of vena cava inferior and EF -laboratory tests -Hemodynamic measurements by PiCCO catheter	40% of oxygen by mask -infusion of crystalloids, noradrenalin, -glucocorticoids iv. -imipenem iv. -CVVH and Cyrosorb terminated
14.10.2015	Oriented, breathing spontaneously, afebrile, blood pressure was 120/100 mmHg, puls 120–100/min, diuresis 200 ml/24 h, SOFA score 7	-pulse oximetry -continuous ECG, arterial blood pressure, intermittent central venous pressure monitoring and hourly urine output -ultrasound of vena cava inferior and EF -laboratory tests -Hemodynamic measurements by PiCCO catheter Chest radiogram	40% of oxygen by mask -infusion of crystalloids, -imipenem iv.
15.10.2015	Oriented, breathing spontaneously, afebrile, blood pressure was 140/100 mmHg, puls 110/min, diuresis 690 ml/24 h	-pulse oximetry -continuous ECG, arterial blood pressure, intermittent central venous pressure monitoring and hourly urine output -ultrasound of vena cava inferior and EF -laboratory tests -Hemodynamic measurements by PiCCO catheter	40% of oxygen by mask -infusion of crystalloids, -imipenem iv. -CVVH
16.10.2015	Oriented, breathing spontaneously, sub febrile, blood pressure was 150/90 mmHg, puls 100–120/min, diuresis 4455 ml/24 h	-pulse oximetry -continuous ECG, arterial blood pressure, hourly urine output -laboratory tests -Hemodynamic measurements by PiCCO catheter	−35% of oxygen by mask -infusion of crystalloids, -imipenem iv. -CVVH intermittently
17.10.2015	Oriented, breathing spontaneously, afebrile, blood pressure was 140/70 mmHg, puls 95–110/min, diuresis 6870 ml/24 h	-pulse oximetry -continuous ECG, arterial blood pressure, hourly urine output -ultrasound of vena cava inferior -laboratory tests	31% of oxygen by mask -infusion of crystalloids, -imipenem iv. -CVVH intermittently
18.10.2015	Oriented, breathing spontaneously, afebrile, blood pressure was 150/80 mmHg, puls 90–110/min, diuresis 4540 ml/24 h	-pulse oximetry -continuous ECG, arterial blood pressure, urine output per day -laboratory tests	3 l of oxygen by nasal cannula -infusion of crystalloids, -imipenem iv. -CVVH intermittently
19.10. 2015	Oriented, breathing spontaneously, afebrile, blood pressure was 160/80 mmHg, puls 70–80/min, diuresis 4000 ml/24 h	-pulse oximetry -continuous ECG, arterial blood pressure, urine output per day -laboratory tests	1 l of oxygen by nasal cannula -infusion of crystalloids, -imipenem iv. -CVVH intermittently -transferred to nephrology ward

Legend: *ICU* intensive care unit, *ECG* electrocardiogram, *EF* ejection fraction, *PiCCO* Pulse Contour Cardiac Output, *CVVH* continuous veno-venous hemofiltration

For regeneration of the kidney function the patient received CVVH intermittently for another 21 days. She was discharged from ICU after 10 days and from the hospital after 76 days.

## Discussion and conclusions

The case describes our clinical experience with the use of hemoadsoption membrane CytoSorb® in combination with CVVH in a patient with Streptococcal pneumonia, complicated by sepsis and septic shock. We used hemoadsoption as an adjunctive therapy and in combination with CVVH in order to control the release of cytokines in a hemodynamically unstable patient.

In one systematic review of human studies on techniques of extracorporeal cytokine removal, standard or ultrafiltration techniques appeared to be inefficient or unreliable in removing cytokines even when coupled with high volume hemofiltration. In the same systematic review adsorption techniques showed promising results based on percentage of cytokins removed, although the data were limited [11]. A small study on 13 critically ill patients with severe sepsis and septic shock, showed an increased transcriptional activity of IL-6 during CVVH, suggesting an immunomodulatory effect during CVVH [12].

Blood purification techniques optimise circulating blood volume and plasma colloid osmotic pressure, correct electrolyte disorders, azotaemia and concomitant metabolic acidosis, decrease body temperature and therefore improve hemodynamic situation. Some decreases in lactate levels, in temperature and in inflammatory marker can be observed as well [11].

In our case we observed significant improvement after CVVH combined with hemoadsorption in echocardiographic measurements of EF (an increase from 20 to 45%) and PiCCO catheter measurements (CI increased from 3.3 L/min/m^2^ to 3,95 L/min/m^2^ and SV from 50 ml to 61 ml). In addition, there was a decrease in the need of vasoactive agents to maintain normotension and adequate cardiac function. We also observed that IL-6 level dropped (from 114 pg/ml to 14,2 pg/ml).

We observed that CI measurements in our patient underestimated the cardiac function, what is not surprising as it depends on both - heart rate and stroke volume. EF measurements by echocardiography more adequately reflected cardiac systolic function, what was already demonstrated by other authors [2].

Clinical studies stress the importance of early treatment by hemoadsoption membranes such as CytoSorb®, in particular within 24–48 h after admission in order to diminish the cytokine storm, responsible for hemodynamic instability in septic shock patients [9]. One of the reasons to start CytoSorb® therapy 36 h from admission was the worsening difficult-to-manage gram-positive septic shock in our splenectomised patient, where the risk of mortality is significantly increased [13].

The reason to continue carbapenem therapy was the fulminant course of infection with septic shock in splenectomised patient. In addition, we only obtained positive pneumococcal urine antigen, but no other specific microbiological cultures to confirm pneumococcal infection.

Transient and significant systolic myocardial dysfunction of the left ventricle (EF 20%) in our septic patient suggested the presence of septic cardiomyopathy as the consequence of increased production of cytokines in a fulminant gram-positive infection [1, 2, 4].

In our patient, we used low dose dobutamine, instead of levosimendan. “Surviving Sepsis Campaign” in 2016 recommends dobutamine as the first line inotropic agent. However, levosimendan increases cardiac myocyte calcium responsiveness and opens ATP-dependent potassium channels, that could exert a more beneficial effect in sepsis-induced myocardial depression [14]. However, due to its higher risk of supraventricular tachyarrhythmia in our patient who was already in sinus tachycardia with the heart rate of 120–130/min, we did not use it. In addition, its long half-life of several weeks limits the practicality of its use in acute shock states [15].

The normal ECG, the absence of chest pain and normal troponin I levels excluded myocardial ischemia as the cause of systolic dysfunction. In our patient, the beneficial effect of CVVH, combined with hemoadsorption, also confirmed a rapid drop in serum lactate, reflecting the reversal of anaerobic metabolism, specific for severe septic shock. This finding is consistent with other studies [16, 17].

Our case suggests that early removal of inflammatory cytokines enables a significant reversal of circulatory failure and in addition, a significant improvement of left ventricular systolic function of septic cardiomyopathy, resulting in improved hemodynamic status, lactacidosis and clinical outcome in septic patients.

## Abbreviations

CI: Cardiac index
CRP: C-reactive protein
CVVHD: Continuous veno-venous hemofiltration
ECG: Electrocardiogram
EF: Ejection fraction
ELWI: Extra vascular lung water index
GEDI: Global end-diastolic index
ICU: Intensive care unit
IL: Interleukins
NT-proBNP: N-terminal-pro brain natriuretic peptide
PiCCO: Pulse Contour Cardiac Output
SatO2: Oxygen saturation
SVRI: Systemic vascular resistance index
TNF-α: Tumor necrosis factor-α
